# Unraveling the mechanisms behind joint damage

**DOI:** 10.7554/eLife.89778

**Published:** 2023-06-27

**Authors:** Wenyu Fu, Chuan-ju Liu

**Affiliations:** 1 Department of Orthopaedic Surgery, New York University Grossman School New York United States; 2 Department of Orthopaedics and Rehabilitation, Yale University School of Medicine New Haven United States

**Keywords:** toll-like receptor 2, sialylation, osteoclast, fusion, rheumatoid arthritis, Mouse

## Abstract

A subtype of myeloid monocyte mediates the transition from autoimmunity to joint destruction in rheumatoid arthritis.

**Related research article** Zhang W, Noller K, Crane J, Wan M, Wu X, Cahan P, Cao X. 2023. RANK^+^TLR2^+^ myeloid subpopulation converts autoimmune to joint destruction in rheumatoid arthritis. *eLife* **12**:e85553. doi: 10.7554/eLife.85553.

Rheumatoid arthritis is an inflammatory autoimmune disease in which the immune system gradually destroys the lining of the joints, leading to pain, swelling and stiffness in the affected regions (Komatsu and Takayanagi, 2022; Weyand and Goronzy, 2021). A key part of disease progression is the transition from autoimmunity to the destruction of the joint. In this process, osteoclasts – cells that break down bone to maintain joint homeostasis – become overactive, leading to the destruction of healthy bone tissue (Fu et al., 2021; Meehan et al., 2021). A complex network of signaling mechanisms drives the formation of osteoclasts from the fusion of immune cells called monocytes. Autoimmunity increases the expression of key molecules in this process, which is thought to drive formation of mature osteoclasts and further joint destruction.

There is currently no cure for this condition, and it remains unclear what exactly triggers the immune system to attack the body, and how this leads to the progressive destruction of the joints. Understanding the mechanisms leading to joint deterioration is thus crucial for the development of effective treatments for rheumatoid arthritis (Fearon et al., 2022; Tang et al., 2011).

Now, in eLife, Xu Cao and colleagues at the Johns Hopkins University School of Medicine – including Weixin Zhang and Kathleen Noller as joint first authors – report that a specific subpopulation of monocytes drives the transition from autoimmunity to joint destruction in rheumatoid arthritis (Zhang et al., 2023).

Using a widely accepted mouse model of rheumatoid arthritis, Zhang et al. carried out a technique called single-cell RNA sequencing to take a closer look at the signaling pathways underlying this change. The experiments revealed that monocytes in these mice overexpress two receptor proteins, TLR2 and RANK, which are important for recognizing foreign substances and initiating an immune response, and for the development of osteoclasts.

Moreover, fluorescent imaging showed increased sialylation, the addition of sialic acids, at specific sites on TLR2 in these RANK^+^TLR2^+^ cells. This was driven by increased expression of enzymes responsible for sialylation in monocytes.

When either the enzymes or TLR2 were experimentally blocked, the monocytes did not transform to become osteoclasts. Consequently, the bone was not resorbed, and the destruction of the joints was mitigated (Figure 1). These findings further support the results of recent research suggesting that the sialylation of TLR2 has a role in initiating cell fusion during osteoclast formation (Dou et al., 2022).

**Figure 1.**
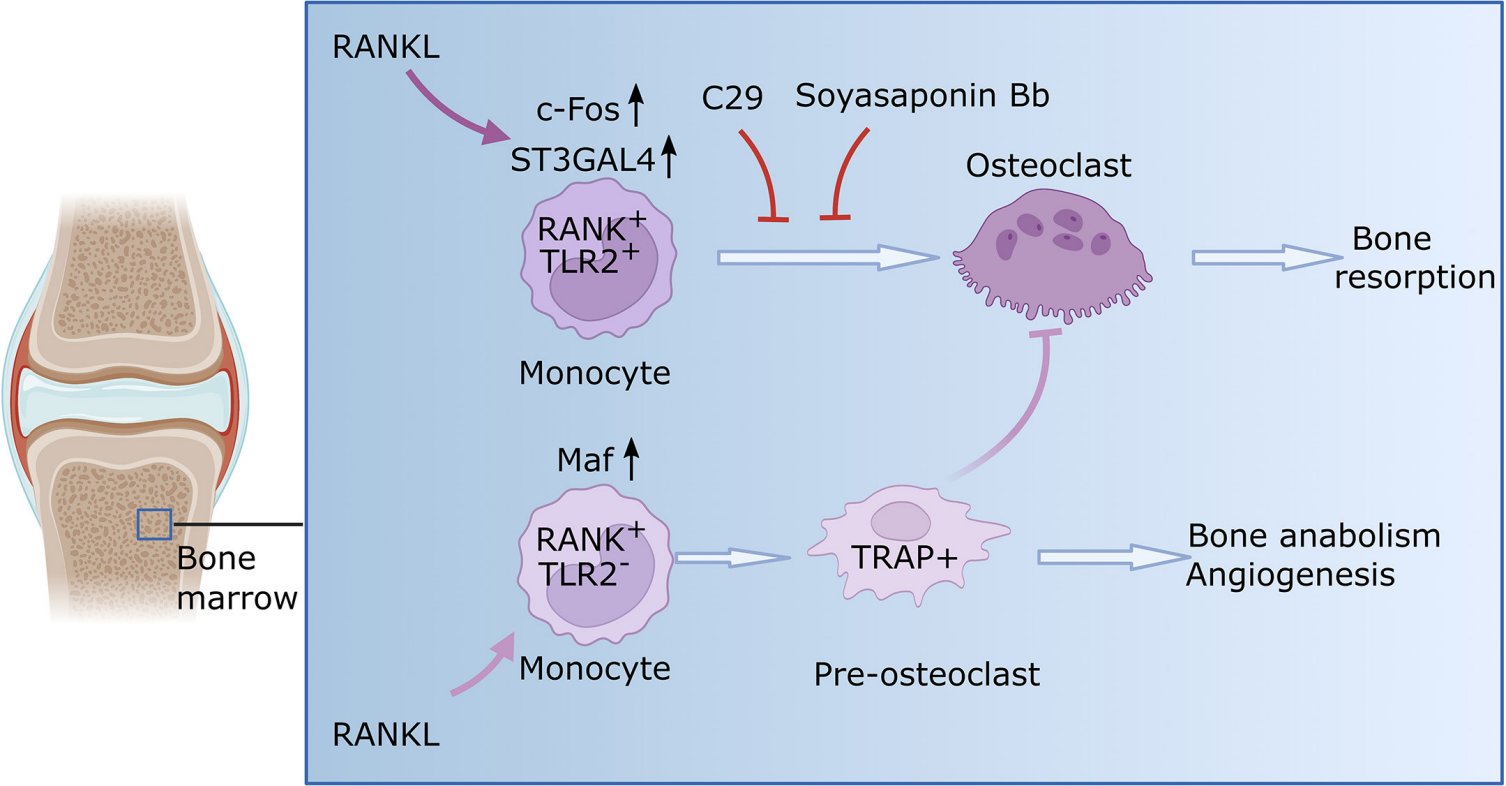
Osteoclast formation in the context of rheumatoid arthritis. Monocytes are a type of immune cell (light purple shape) that can develop into osteoclasts (dark purple shape), cells that can degrade healthy bone to initiate remodeling. In certain conditions, such as rheumatoid arthritis, the activity of osteoclasts is increased, which leads to progressive bone loss. Zhang et al. identified two subsets of monocytes. One subset (RANK^+^TLR2^+^) overexpressed two relevant signaling molecules, RANK and TLR2, and is involved in bone resorption. When RANK is activated by RANKL in this subset, the enzyme responsible for TLR2 sialylation (ST3GAL4) becomes upregulated through a molecule called c-Fos (a transcription factor critical for the regulation of osteoclast differentiation). Consequently, TLR2 undergoes sialylation, a process that adds sialic acids that enable TLR2 to bind with different molecules in order to allow fusion of cells. Blocking sialyation or TLR2 with specific molecules (C29 and soyasaponin Bb, respectively) prevents mature osteoclasts from forming and the bone remains intact. The other monocyte subset (RANK^+^TLR2^-^) exhibited high levels of RANK but low levels of TLR2. In this subset, RANKL prompts the monocytes to differentiate into pre-osteoclasts that express a molecule called TRAP+, but they fail to mature into fully-functional osteoclasts. Instead, these cells have anabolic properties and promote the production of new bone and blood vessels. This figure was generated using BioRender.com.

Single-cell RNA sequencing further revealed a previously unknown subset of myeloid monocytes, which overexpress RANK but do not express TLR2. These RANK^+^TLR2^-^ monocytes were able to differentiate into precursor osteoclasts, but they were unable to fuse and mature into fully functional osteoclasts (Figure 1). They promoted, however, the expression of proteins that further the formation of new blood vessels and bones, which may explain presence of precursor osteoclasts displaying anabolic properties in bone (i.e., with the ability to promote the biosynthesis that further tissue growth).

Zhang et al. provide crucial insights into the mechanisms driving the transition from the onset of autoimmunity to joint destruction in rheumatoid arthritis. The findings emphasize the central role of myeloid monocytes that overexpress both RANK and TLR2, and the sialylation of TLR2 in driving the fusion of monocytes and consequent resorption of bone. Targeting the sialylation process in these monocytes represents a promising avenue for preventing autoimmune-mediated joint destruction and preserving bone health in patients with rheumatoid arthritis, and potentially other autoimmune disorders. Moreover, the identification of monocytes that lack TLR2 offers valuable insights into the complex cellular dynamics involved in joint destruction and may open new avenues for exploring the potential of these cells in promoting bone repair and regeneration. More research is needed to better understand if and how the two monocyte subsets interact and their clinical relevance in humans.

In summary, the potential therapeutic targets identified in this study should help with efforts to develop drugs that prevent autoimmune-mediated joint destruction and promote bone production in rheumatoid arthritis and various autoimmune diseases.
